# A Systematic Review of Methods to Improve Attitudes Towards Childhood Vaccinations

**DOI:** 10.7759/cureus.5067

**Published:** 2019-07-02

**Authors:** Rawan Nour

**Affiliations:** 1 Miscellaneous, College of Health Solutions, Arizona State University, Phoenix, USA

**Keywords:** anti-vaccination movement, vaccination, childhood

## Abstract

Although measles was eradicated in the United States in 2000, there has been an increasing number of cases. As of April 4, 2019, the Centers for Disease Control and Prevention (CDC) reported 465 cases of measles as compared to 372 in 2018. Pockets of unvaccinated communities and travelers bringing measles from countries with large outbreaks are attributed to the rise in cases in the United States. With the increasing anti-vaccine sentiment, it’s imperative to take preventative measures to avoid the proliferation of deadly diseases.

The objective of this study was to determine effective techniques to decrease vaccine refusal and increase the childhood vaccination rate. To this effect, a systematic review of English peer-reviewed articles published in the years 2000 to 2019 using Arizona State University’s online database was conducted. The titles, abstracts, and discussions for each journal article were screened for methods that promote vaccination. Any articles that included vaccine development, promoting vaccination coverage rate in developing countries, pregnant women, healthcare workers, and animals, and promoting the uptake of vaccines that aren’t part of CDC’s seven-vaccine series were excluded.

A total of nine journal articles were identified, including five systematic reviews, one case study, a randomized controlled trial, a literature review, and a quasi-experimental study. The methods discovered pertained to three themes: technological, mass marketing or campaigning, and direct communication. The Guide to Tailored Immunization Programme (TIP) in conjunction with visually enhanced educational materials and storytelling articles were found to be effective tools in encouraging vaccination coverage.

The basis of any strategy is determining the perspective and needs of the target population and tailoring the approaches to match them to alleviate barriers that hinder vaccination uptake. The use of technology perpetuates the efficacy of social marketing strategies, further promoting vaccines and their benefits.

## Introduction and background

Introduction

Measles Outbreaks in the United States

Measles is an airborne disease that spreads through the air, as droplets of viral nuclei when a person coughs or sneezes. Before the measles vaccination program, there were “approximately 500,000 cases reported each year that resulted in 400 to 500 deaths, 48,000 hospitalizations, and 1,000 cases of encephalitis.” The program resulted in more than a 99% reduction in measles cases [1]. In 1978, the CDC set a goal to eradicate measles by 1982, which refers to the “absence of continuous” measles “transmission for greater than 12 months,” but they didn’t meet their goal. In 1989, there were measles outbreaks “among vaccinated school-aged children,” so the Advisory Committee on Immunization Practices (AICP), the American Academy of Pediatrics (AAP), and the American Academy of Family Physicians (AAFP) recommended a second dose of the measles-mumps-rubella (MMR) vaccine for all children contributing to a higher declination in reported cases. In 2000, measles was eradicated in the United States [2].

**Figure 1:**
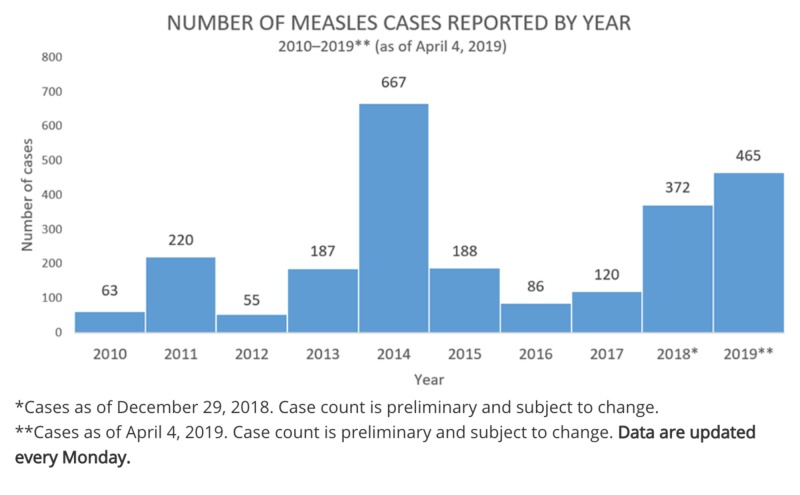
A bar graph from the Centers for Disease Control and Prevention (CDC) website showing the number of measles cases from 2010 to April 4, 2019.

As of April 4, 2019, the Centers for Disease Control and Prevention (CDC) reported that there are individual cases of measles confirmed in 15 states, resulting in a total of 465 reported cases as compared to 372 in 2018 (Figure 1) [3-4]. The outbreaks are attributed to unvaccinated travelers bringing measles back from Israel, Ukraine, and the Philippines, where there are large measles outbreaks [3]. The revival of measles raises concerns surrounding the welfare of adults and children and emphasizes the importance of vaccinations.

Childhood Vaccinations

According to a study conducted by the Blue Cross Blue Shield Association, early childhood vaccination rates have been steadily increasing. However, a wide variation in vaccination rates exists between states and local communities in the United States. The study examined children born from 2010 and 2013 and determined the vaccination rates three years after their birth year, which is CDC’s suggested completion date for the seven-vaccine series, which include DTaP (diphtheria, tetanus toxoids, and acellular pertussis), polio (poliovirus), MMR (measles-mumps-rubella), Hib (Haemophilus influenzae type b), HepB (hepatitis B), VAR (varicella), and PCV (pneumococcal conjugate) vaccines [5-6].^ ^They’ve found that among the four cohorts, 23% are under-vaccinated. Several factors contributed to under-vaccination in 2016, including missed well-child visits, refusal, inferred delays, and other unidentified reasons. The largest percentage of under-vaccinated children is due to missed well-child appointments, which is 62%. Inferred delays refer to delays in scheduling the seven-vaccine series, and they account for 4% of under-vaccinated children. Vaccination refusals make up 6%, which comparatively doesn’t seem like a substantial amount, but there has been approximately a 70% increase in vaccination refusal “for children born in 2013 as compared to children born in 2010.” Parents or guardians who refused vaccination, refused all of them, suggesting that one vaccine isn’t more likely to be refused than the other [5]. Additionally, the considerable jump in vaccination refusals emulates a deep-rooted problem that will result in life-threatening consequences.

Anti-Vaccination Propaganda

The anti-vaccination propaganda isn’t a new concept, as evidenced by Dr. Andrew Wakefield’s publication in 1998 that falsely linked autism to the measles-mumps-rubella (MMR) vaccine [7]. But with the rise of social media, the anti-vaccine sentiment has proliferated. Although social media has played a pivotal role in how biomedical and healthcare companies communicate with their consumers and match the consumers’ “needs and interests with available products and services,” it has also provided a platform that allows consumers to spread misinformation. Social media has enabled consumers to “rapidly seek health information, share medical advice, AND directly manage health conditions,” all of which could be considerably beneficial but only with caution [8]. According to the National Assessment of Adult Literacy (NAAL), only “12 percent of U.S. adults have proficient health literacy” while approximately 35% have basic or below-basic health literacy skills. The limited literacy skills render them incapable of thoroughly understanding available health information to use appropriately, which further propagates misinformation [9].

The nineteenth century marks the beginning of the compulsory vaccine era. It began with the Dutch government in 1871 where they required that all schoolchildren receive vaccinations to prevent the propagation of smallpox. But in 1881, it came to a halt when the Association to Oppose Compulsory Vaccination was established. The association presumed that mandatory vaccinations violated individuals’ liberties and, in response, promoted religious exemptions. In 1879, the Anti-Vaccination Society of America was founded, and its leaders were worried that government intervention would threaten their practices and manufacturers feared that it would limit their trade. Albeit the compromised stance of the leaders of the Anti-Vaccination Society of America, many states repealed compulsory vaccinations. In 1889, the Royal Commission on Vaccination was established to retaliate the rise of the anti-vaccination movement, and in 1907, it passed laws to allow individuals to opt out as a compromise. With that, the anti-vaccination sentiment diminished progressively, more so when medical licensure laws became more structured and when the federal and state government gained more control over public health. The movement was originally against the compulsory nature of vaccinations “rather than vaccination” itself, but its current reemergence is due to concerns about vaccine safety. Unlike the nineteenth and twentieth centuries, the anti-vaccination movement gained deep-rooted traction with the existence of social media and negative connotations surrounding immunizations perpetuated exponentially [10].

Commitment to vaccinations follows the continuum model whereby the intermediates and extremes are represented. Vaccine hesitancy is the manifestation of the intermediates and includes behavioral strategies used to seek alternate vaccination schedules, such as selective refusal, delay of certain types of vaccines, or beginning vaccinations later in a child’s life [11].^ ^The lack of confidence in vaccine safety and efficacy along with the quality and convenience of vaccination services contribute to the likelihood an individual will get or have their children vaccinated [12]. Three common false immunological claims influence the public to be more reluctant to get vaccinations: antigenic overload, prominence of autoimmune disorders, and natural immunity. The antigenic overload claim alleges that children’s immune systems are incapable of handling the number of antigens given in immunizations. In Wisconsin, 64.9% of 236 parents that claimed non-medical exemptions stated that they are concerned too many immunizations could weaken their children’s immune system. Those who concur with the antigenic overload claim argue that too many vaccinations could trigger a “cytokine storm,” which, in turn, would result in adverse health events. Although the claim hasn’t been substantiated using scientific evidence, there is a rare occurrence that antigenic simulation caused by vaccines could result in an anaphylactic response. Additionally, there are four significant articles of evidence that antagonize this claim. First, the amount of antigens from micro-organisms that are exposed to children the moment they’re born exceeds the amount and variety of antigens given via vaccines. Second, “studies of vaccine efficacy and safety include” combinations of the different vaccines in the seven-vaccine series didn’t establish any evidence of symptoms and signs of antigenic overload. Third, vaccinated children haven’t demonstrated evidence of antigenic overload. Fourth, throughout the years, the amount of antigens in vaccines has drastically decreased with the implementation of a vaccine schedule and improvements in vaccinations. All four declarations augment the fact that antigenic overload shouldn’t be a concern for parents and that there is sufficient evidence to combat any statements indicating it should be a reason to refuse immunizations. The second claim that anti-vaccinationists have promoted is that vaccines can cause autoimmune diseases. The claim suggests that the vaccine components mimic a human protein so much so that when antibodies are produced, they bind to human analogs instead of the antigens they’re meant to attach to, resulting in an autoimmune response. To identify any possible associations between vaccines and autoimmune diseases, specifically type I diabetes mellitus, multiple sclerosis, and Guillain-Barre syndrome, the Institute of Medicine organized a panel of experts to review more than 12,000 published reports and failed to find any association. The third claim asserts that “immunity induced by ‘natural infection’ is safer than vaccine-induced immunity.” Although acquiring the infection could result in “superior immunity,” acquiring the infection could impose high risks of lethal or disabling complications. Additionally, booster shots, which are subsequent shots of the same vaccine, strengthen the immunological response to certain diseases without the risks of death or other complications. Further, immunocompromised or immunosenescent individuals are unable to retrieve live viral vaccines and depend on others that are capable to do so to protect them from a natural infection [13]. All claims made by those who argue against vaccinations have been countered via peer-reviewed studies, but its advancement debilitates the effect that vaccinations have done on public health thus far.

The Impact of Psychosocial Theories and Models of Behavior on Vaccination Uptake Rates

The greatest concern surrounding the spread of misinformation, particularly around vaccinations, is its impact on behaviors. The health belief model (HBM) identifies “two types of health beliefs” that contribute to the likelihood an individual will respond to an illness: “perceptions of the threat of illness and the evaluation of the effectiveness of behaviors to counteract this threat.” If an individual finds that they are highly “susceptible to a particular condition or illness” and believe undertaking a behavior to counteract it outweighs the costs of the condition or illness, then they will engage in that behavior. The triggers that motivate an individual to take action could be internal (e.g., symptoms) or external and are called cues to action. In the matter of vaccinations, if an individual finds that they are highly susceptible to a disease that a vaccine prevents and they find that the risks associated with the disease outweigh risks associated with vaccines, then they’ll retrieve vaccinations. Many diseases that were widely prevalent before the vaccination program have been eradicated, so individuals may feel that the likelihood of contracting the disease is low. In turn, they may perceive the risk associated with vaccines as artificially higher than contracting the disease and become complacent [14]. The decreased prevalence of the disease is due to the large percentage of the population being vaccinated, which contributes to herd immunity. Herd immunity refers to the protection that unvaccinated and immunocompromised individuals receive when a large portion of the population is immunized, which reduces the transmission rate of a disease significantly [15]. The potency of herd immunity dwindles as the vaccination retrieval rate decreases, making it essential for individuals to get vaccinated irrespective of the disease’s pervasiveness continuously.

In addition to the HBM, the theory of planned behavior (TPB) and the social cognitive theory (SCT) establish a relationship between internal and external factors and behavior. The TPB identifies two determinants of behavior: “intentions to engage in that behavior and perceived behavioral control (PBC) over that behavior.” An individual’s attitude, subjective norms, and PBC influence intentions and are influenced by underlying beliefs. PBC refers to the availability and accessibility of tools needed to perform the behavior successfully. The likelihood that an individual will pursue a behavior increases as the perceived outcome and subjective norms are more positive and as PBC increases. SCT recognizes four factors that determine behavior: goals, outcome expectancies, self-efficacy, and sociostructural variables. SCT is comparable to the TPB. Self-efficacy and PBC are analogous as are sociostructural variables and subjective norms. Both self-efficacy and PBC indicate that the likelihood an individual pursues a behavior is dependent on the perceived probability that an individual will be successful in engaging in a specific behavior. Additionally, both sociostructural variables and subjective norms refer to the environmental elements that either promote or hinder the behavior. The three different doctrines highlight how various aspects of an individual’s life impact his or her behavior. Vaccine refusal and hesitancy is due to a myriad of environmental and internal factors, and to counteract it and similar behaviors, they must be identified and analyzed [14].

The epidemiological triad highlights three determinants of vaccine hesitancy among parents: environmental, vaccine-specific, and parental-specific factors. Environmental factors include the patient-health professional relationship, social norms, and collective values, school immunization requirements, media and communication, and vaccine policies. The relationship between a patient and physician must foster trust and solidarity to encourage families to accept vaccines. According to a review conducted on 12 research articles, nurses that are vaccinated and promote vaccination are proportionate to the level of motivation in patients. Patients are less likely to vaccinate themselves or their children if health professionals aren’t doing it themselves; thus it’s imperative for health professionals to lead by example. Patients rely on their health professionals to provide exceptional care and reliable health information, so having a clear understanding of the current vaccine schedule and ratiocinative and sensible communication skills is fundamental. School immunization requirements and public health vaccine policies quadrate. Both educational and public health institutions have a role in addressing the concerns surrounding vaccinations and in educating them in collaboration with health professionals. Schools may be the only source of health information for those that don’t have access to health care, so they may even be more integral in advocating for vaccines. The collaboration between health professionals and public health and educational institutions could instill social norms and collective values that encourage individuals to be compliant and get vaccinations, even if there is misinformation or sensationalized vaccine injuries circling media and communication outlets. Vaccine-specific factors refer to vaccine efficacy and safety and disease susceptibility perception. The more parents believe that vaccines are safe and effective and that they are susceptible to acquiring the disease, the more likely they’ll retrieve vaccinations for themselves and their children. Finally, patient-specific factors include demographics and the parent’s decision, knowledge, and past experiences. There has been no evidence that supports the association between racial groups and vaccination uptake rates, but there has been evidence of an association between the level of parental education and immunization coverage. Parents with a lower educational level are more likely to be vaccine-hesitant. The information that they are exposed to about vaccines and their effects could be limited, and they are less likely to seek out alternative sources [12]. The epidemiological triad illustrates the application of the HBM, SCT, and TPB, further acknowledging the importance of an interdisciplinary approach to alleviating anti-vaccine sentiments and vaccine hesitancy.

Having a thorough understanding of what vaccine-hesitant or anti-vaccinationists believe about vaccines and what impacts an individual’s behavior is fundamental for creating and compiling strategies to address anti-vaccine sentiment. I have conducted a systematic literature review of strategies or concepts that could be used to improve vaccination beliefs and, in turn, immunization rates among children.

## Review

Methods

Search Methodology

A systematic literature review was performed to determine effective strategies that could be implemented to encourage families to vaccinate their children. The search was limited to English, patient-centric, peer-reviewed journal articles published between the years 2000 and 2019. Additionally, articles that included vaccine development, methods for promoting any vaccines that aren’t part of CDC’s seven-vaccine series schedule, vaccination coverage rates in developing countries, animals, healthcare workers, and pregnant women, and any duplicate or similar studies were excluded. The following search words, phrases, and combinations of each were used: improving, anti-vac*, vaccination beliefs, addressing vaccine hesitancy, vaccine-preventable disease, improving or tackling anti-immuniz*, strategies, overcoming barriers to vacc*, childhood vaccinations, and addressing anti-vaccination beliefs. Arizona State University’s online database, which compiled relevant articles from the following databases: ProQuest, Scopus (Elsevier), EBSCO, PubMed, and ScienceDirect (Elsevier), was used.

Study Selection and Data Collection Process

The titles, abstracts, and conclusions of peer-reviewed journal articles were examined to determine suitability for the proposed objective. Sources that indicated evidence of studies conducted to identify novel or widely accepted methodologies that could be used to public health vaccinations and sources that identify reasons for vaccine hesitancy and/or refusal and propose ways to overcome it were chosen. The types of studies used include literature and systematic reviews, randomized controlled trials, and quasi-experimental and case studies. Overarching themes were detected while screening studies and they were categorized to epitomize the approaches that could be made to address anti-vaccine sentiment and vaccine hesitancy. To assess the risk of bias, the studies’ methods, results, and discussions sections were inspected.

Results

**Figure 2:**
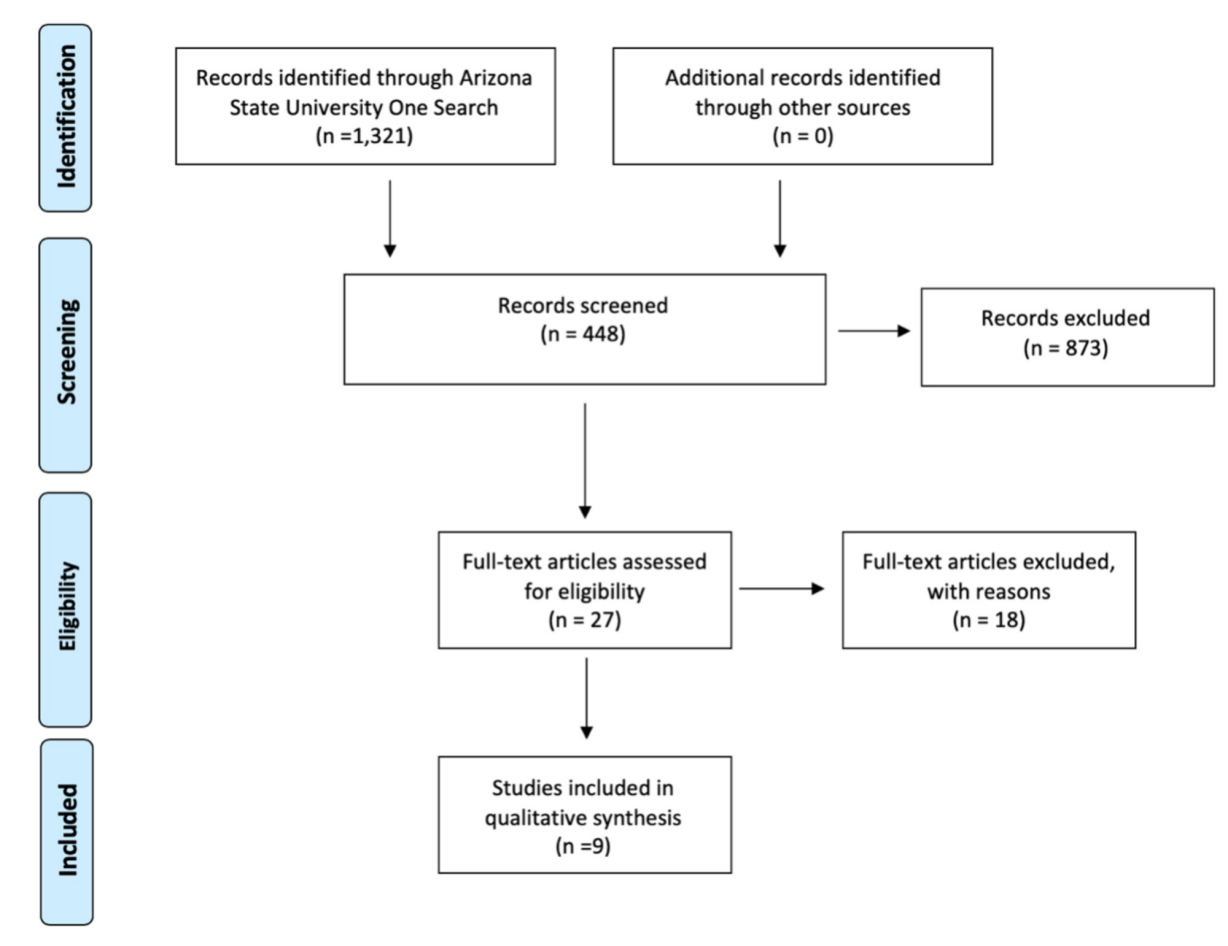
A PRISMA 2009 flow diagram depicting the number of studies included in the qualitative synthesis using the mentioned inclusion and exclusion criteria.

A total number of nine peer-reviewed articles were used to address anti-vaccine sentiment and vaccine hesitancy (Figure 2). There were three motifs present among the articles used: technological, mass marketing or campaigning, and direct communication strategies (Table 1).

**Table 1:** Characteristics of studies included in the qualitative synthesis

Characteristics of Studies Included in the Qualitative Synthesis^a^		
Theme	Type of Study	Article Name
Technological	Case Study	Lessons from an Online Vaccine Communication Project
	Systematic Review	Opportunities and Challenges of Web 2.0 for Vaccination Decisions
	Systematic Review	Opportunities for Utilizing New Technologies to Increase Vaccine Confidence
	Systematic Review	Utilizing Health Information Technology to Improve Vaccine Communication and Coverage
Mass Marketing or Campaigning	Systematic Review	Addressing Vaccine Hesitancy: The Potential Value of Commercial and Social Marketing Principles and Practices
	Systematic Review	Diagnosing the Determinants of Vaccine Hesitancy in Specific Subgroups: The Guide to Tailoring Immunization Programmes (TIP)
	Randomized Controlled Trial	Misinformation Lingers in Memory: Failure of Three Pro-Vaccination Strategies
Direct Communication	Literature Review	Addressing Barriers to Vaccine Acceptance: An Overview
	Quasi-Experimental	Increasing Immunization Adherence among Infants of Low-Income Parents: The Effects of Visually Enhanced Education
^a^A total of nine peer-reviewed articles was included in the qualitative synthesis, and three themes were identified: technological, mass marketing or campaigning, and direct communication strategies.		

Technological

Four journal articles evaluated and recommended technological strategies, including one case study and three systematic reviews (Table 1). All four articles identified the importance of understanding the climate of vaccination attitudes and the characteristics of the individuals that are vaccine-hesitant or are anti-vaccinationists, including their demographic and socioeconomic status and geographical locations. Additionally, all articles analyzed identified social media platforms and websites as a modality to promote vaccination coverage [16-19]. Articles by Finnegan et al. and Cornelia et al. determined that story-telling articles and answers to frequently asked questions on websites were found to be effective and were more attractive to those seeking vaccine information [16-17]. The case study conducted by Finnegan et al. analyzed the effects of a vaccine information website created by Vaccines Europe called Vaccines Today on vaccination attitudes. The case study determined the efficacy of the website’s content by analyzing the time spent on different webpages. In addition to the frequently asked questions and story-telling articles, they found that videos with animations were also a compelling tool [16]. A systematic review conducted by Nowak et al. identified health information technology as a tool to remind patients and clinicians of the types of vaccinations needed at appointments and to streamline and improve workflow and vaccine documentation [18]. Articles by Finnegan et al. and Wilson et al. identified privacy concerns and lack of dissemination as limitations of the efficacy of technological tools on immunization promotion [16,19].

Mass Marketing or Campaigning

Three journal articles proposed and analyzed mass marketing or campaigning strategies including one randomized controlled trial and two systematic reviews (Table 1). All articles emphasized the importance of understanding the population before implementing marketing or campaigning strategies and identified social marketing as an effective tool for vaccine promotion. Furthermore, all articles acknowledged that successful campaigns are tailored to different subgroups of the population at different times and continuously [20-22]. The journal article authored by Butler et al. introduced the Guide to Tailoring Immunization Programmes (TIP), which uses social marketing principles and behavioral model framework to identify behavioral determinants of vaccine hesitancy and, consequently, propose interventions [20]. A randomized controlled trial conducted by Pluviano et al. using a convenience sampling method randomly sampled 134 college students to assess the effectiveness of three commonly used strategies in vaccine promotion: fear appeal, icon/fact box, and myth versus facts. Fear appeal contained images of those inflicted by VPD, icon/fact box displayed a table with the possible side effects of measles and MMR vaccine, and the myth versus facts sheet addressed the misconception with the corresponding evidence-based vaccine fact. All strategies were found to be ineffective, with the icon/fact box being the least ineffective. The article concluded that the tailored approach accounts for attitudes, social norms, standards, and structural barriers and storytelling as modalities that are effective and portray accurate and comprehendible vaccine information, which, in turn, promote vaccine coverage [21]. Nowak et al. wrote a systematic review that also identified fear appeal as a common strategy but didn’t evaluate its efficacy [22]. Although limitations and biases were identified in the randomized controlled trial article, such as the inability to generalize data found due to the limited demographic and the use of convenience sampling, the results uncovered were bolstered by the article that identified it as a common marketing strategy [21-22]. The articles by Butler et al. and Nowak et al. identified the inability to apply the strategies to all behaviors as limitations to their propositions [20-21].

Direct Communication

Two journal articles proposed and analyzed direct communication strategies, including one quasi-experimental study and one literature review. Both articles highlighted the significant role that healthcare providers play in addressing vaccine hesitancy and anti-vaccine sentiment and the importance of fostering trust and a healthy relationship between the patient and clinician [23-24]. The quasi-experimental study conducted by Papchrisanthou et al. evaluated the immunization adherence rates between two groups of low-income parents with four to 14-day-old infants. One group received verbal and visually enhanced education (VEE) containing five images of children inflicted by vaccine-preventable disease (VPD) at the “child’s initial visit and again during the one-month office visit.” The other group served as the control group and received usual care (UC), which contains verbal education acknowledging the potential side-effects of vaccines and VPD and vaccine information sheets. The study found that 68% of participants receiving VEE retrieved all three sets of immunizations, whereas only 33% of UC participants did, indicating statistical significance using an alpha level of 0.05 [23].

Discussion

A combination of technological, mass marketing or campaigning, and direct communication strategies is integral for identifying the root causes of the anti-vaccine sentiment and vaccine hesitancy and for identifying ways to modify the beliefs and promote vaccination coverage. Signs of vaccine hesitancy are obscure but identifying them expeditiously is vital for maintaining vaccine acceptance.

Social Marketing and Vaccine Coverage

Social marketing is the application of marketing principles to influence health behaviors. It utilizes six “Ps” rather than just four as typically seen in general marketing principles: product, price, place, promotion, partnerships, and participation of key stakeholders. Product refers to the features and benefits associated with retrieving vaccinations, price refers to monetary, physical, and temporal costs associated with the behavior, place refers to accessibility, promotion refers to persuasive factors used to promote behavior, and using partnerships, and the participation of key stakeholders refers to establishing public health policies and social norms. After collecting the information pertained to the six “Ps,” brand positioning, tactical segmentation, and immunization convenience are determined. Brand positioning establishes the target population, how public health agencies and healthcare providers want vaccines to be perceived, and the benefits that would need to be highlighted. Tactical segmentation researches and analyzes demographic and psychographic characteristics in addition to attitudes, knowledge, and behaviors to understand how the target population can be persuaded. And immunization convenience indicates cogent methods to reach the target population by identifying positive behavioral determinants that encourage desired behavior that illicit long-term benefits [21]. Auspiciously, the World Health Organization (WHO) Euro Vaccine-Preventable Disease and Immunization (VPI) program developed the 2013 Guide to Tailoring Immunization Programmes (TIP) to define behavioral determinants of vaccine hesitancy and propose interventions. It explicitly “identifies vaccine-hesitant populations, diagnoses the demand and supply-side barriers and enablers for vaccinations in these populations, and designs evidence-based responses to vaccine hesitancy adapted to setting and context.” The TIP uses “social marketing models, behavior insight methods, and the Population Services International (PSI) Delta Social Marketing Process 7 Steps” to compile effective and multifaceted approaches [20]. The use of TIP coupled with the different essential components identified in the various articles assessed like storytelling, visually enhanced educational materials, videos, and presumptive expression of evidenced-based vaccine information could result in the alleviation of vaccine hesitancy and immunization coverage.

Patient-Clinician Relationship

Fostering and maintaining a positive, trusting, and nurturing patient-clinician relationship by using motivational interviewing skills and by merely being honest is fundamental in patients’ compliance with clinical advice, particularly vaccine uptake. And it increases the likelihood that the patient seeks vaccine information from the clinician rather than friends, family, and the Internet [23]. Further, using presumptive approaches, easy-to-understand pictorial materials portraying risk, and loss-framed messages also decrease vaccine hesitancy and increases the prospect that the patient will retrieve vaccinations for themselves and their children [23-24]. The clinician also plays a critical role in identifying any barriers that the patient has including financial and psychosociological. Once the barriers are identified, the information could be collected to determine and compile the population’s characteristics that face the same barriers and, in turn, create a tailored approach using TIP [20].

Technology: Data Collector and Delivery Tool

Web 2.0 refers to the evolution of the Internet from a one-way communication facilitator to a multidimensional one. Its applications allow individuals to create, share, and comment on content creating a platform for expressivity and information. It is manifested into social media websites such as “Facebook, Twitter, Wikipedia, LinkedIn, and YouTube" [17]. As of January 10, 2018, 77% of the United States population own a smartphone, enabling access to virtual interactions and exposure to content and information [25]. This novel innovation presents convenience in spreading accurate and inaccurate information, but with the paucity of numeracy and health literacy skills, misinformation is more prevalent and more memorable. Unfortunately, incorrect information is difficult to correct because it is duly reinforced on a daily basis.

The uses of health information technology are malleable, rendering it a critical tool in collecting and analyzing patient information and in providing clinical decision-support systems that alert both the healthcare provider and patient of upcoming immunizations [18]. It has the potential to construct TIP based on the patient’s characteristics to improve attitudes towards vaccinations and encourage uptake, but its most significant barrier is privacy. With the rise of technology, the general population’s concern with protecting their privacy has risen correspondingly and rightfully so. This accentuates the importance of fostering a trusting relationship between the patient and the physician. Transparency is of the utmost importance in not only motivating vaccination coverage but in also collecting vital information to be able to further medical applications and better the public’s health.

Limitations

In addition to the limitations and biases identified by the journal articles used, the novelty of the topic restricts the accumulation of data to comprehensively assert different tactics used to alleviate vaccine hesitancy and anti-vaccine sentiment. Additionally, the different determinants of behaviors vary widely, and although TIP has been deemed an effective tool for identifying them and proposing appropriate interventions, its efficacy relies on current and a substantial amount of patient information.

## Conclusions

The reemergence of eradicated diseases portrays the impact that social media has on the general population. Collaboration among different disciplines is essential for creating effective strategies that promote public health boundlessly and specifically promote vaccination uptake rates. With the increased prevalence of technology in our world today, we have the elevated ability to influence quickly and more provokingly by understanding the population and using a compilation of proven methodologies like TIP.
